# Disposal practices of unused and expired pharmaceuticals among general public in Kabul

**DOI:** 10.1186/s12889-016-3975-z

**Published:** 2017-01-07

**Authors:** Mohammadk Bashaar, Vijay Thawani, Mohamed Azmi Hassali, Fahad Saleem

**Affiliations:** 1 Health Policy Analyst, SMART Afghan International Trainings & Consultancy, Kabul, Afghanistan; 2 Professor of Pharmacology, People’s College of Medical Sciences & Research Centre, Bhanpur, Bhopal, 462037 India; 3 Professor of Social and Administrative Pharmacy, School of Pharmaceutical Sciences, Universiti Sains Malaysia, 11800 Minden, Penang Malaysia; 4 Faculty of Pharmacy and Health Sciences, University of Baluchistan, Quetta, Pakistan

**Keywords:** Pharmaceutical, Disposal, Unused, Medicine, Expired, Afghanistan, Practices

## Abstract

**Background:**

Most of the medicine users remain unaware about the disposal of unused or expired medicines. The aim of this study was to know the disposal practices of unused and expired medicines among the general public in Kabul.

**Methods:**

This was a descriptive, cross-sectional survey, conducted through face-to-face interviews using prevalidated structured questionnaire. Returned questionnaires were double-checked for accuracy. Statistical Package for Social Science (SPSS) version 23 was used for statistical analysis.

**Results:**

Total of 301 valid questionnaires were returned with a response rate of 100% in which 73.4% men and 26.6% women participated. More than half of the respondents were university graduates. Interestingly, 83.4% of the interviewees purchased medicines on the prescription of which 47.2% were university graduates, while 14.6% purchased medicine over the counter. Among the respondents, 46.5/100 purchased antibiotics and the remaining purchased NSAIDs, anti-hypertensive and anti-diabetic medicines. Significantly, 97/100 checked the expiry date of medicine before buying. Majority (95.3%) of the respondents’ stored medicines at home. 77.7% of the respondents discarded the expired medicines in household trash. Majority of respondents held government responsible for creation of awareness for proper medicine disposal. Almost entire sample (98%) felt that improper disposal of unused and expired medicines can affect the environment and health.

**Conclusion:**

Gaps exist in practices, therefore robust, safe and cost-effective pharmaceutical waste management program supported with media campaign is needed. Healthcare practitioners and community pharmacists should offer training to educate customers on standard medicine disposal practices.

## Highlights

- Disposal practices of unused and expired pharmaceuticals among general public in Kabul, Afghanistan
- We examine the knowledge and practices towards unused and expired pharmaceutical among general public
- Improving the understanding of future medication waste disposal among families
- Increasing information on safe and standard pharmaceutical waste
- Help the policymakers to take firm steps to encourage standard pharmaceutical waste management

## Background

Increasing disease incidence and prevalence necessitate healthcare practitioners to prescribe and dispense different medications. The consumer (patients) are not able to use all the dispensed medications because of adverse effects, alteration of dosage, feeling healthy, medications reaching the expiration date, promotional practices by manufacturers’, physicians’ prescribing practices, or dispensers’ practices [1, 2]. According to World Health Organization (WHO), more than half of all medication is inappropriately prescribed, prescribed and sold, which causes unnecessary storage and creates environmental threat [3]. Non-adherence to medication can also cause storage of left over medicines at home. According to WHO 50% of patients fail to take medicine correctly [4]. Therefore, it is usual that, families and patients are in possession of unused or expired medications and its risks have gained attention across the world [5].

When there is a concern of unused and expired medication storage, patients and family members require clear guidance about its disposal [1]. The presence of unused and expired medications in cabinets and cupboards is a potential threat and can be harmful to humans, environment and wildlife [6–8]. Specifically, the presence of discarded medicines in waterways and drinking water is a serious and multifaceted issue that has gained national and international attention with the public, lawmakers, and regulators [9]. For instance, non-steroidal anti-inflammatory drug (NSAID) diclofenac has been shown to induce renal failure in vultures following the ingestion of carrion from cattle treated with this drug [10]. The improper disposal of unused and expired medication challenges the environment for example in the USA many drugs such as acetaminophen, verapamil, and estradiol are found in waterways. [11]. The trace levels of ethinyl estradiol, the active component of a common oral contraceptive, impairs sexual development and the feminization of fish [12]. Evidence shows that the presence of antibiotics in water may lead to antibiotic resistance [13] and in long term may cause genetic effects in humans and marine life [9]. In Kabul, Afghanistan, the byproducts of a mass vaccination campaign of 1.6 million against polio in October 2008 were discarded in the local municipal waste, causing infectious injury to individuals searching waste dump sites for reusable items. Other medical wastes including pharmaceuticals have been found lying in the open land-fills near hospitals in urban areas [14]. Similarly, it has been found that more than 60 hospitals in Kabul do not have incineration facility or access to other essential Health Care Waste Management (HCWM) equipments [15, 16].

The HCW includes all the waste generated by health-care establishments, research facilities, and laboratories [17]. Pharmaceutical waste is one constituent of the HCW, which contains expired pharmaceuticals or no longer needed, contaminated items, or pharmaceuticals, which need clear and systematic disposal approach to get rid of its hazardous effects.

Therefore, WHO’s European Centre for Environment and Health in France, set up an international working group to produce a practical guide, addressing particularly the problems of HCWM in developing economies [18]. In addition, some programs such as Disposal of Unwanted Medication Properly (DUMP) campaign was launched in New Zealand [19] and in Canada ENVIRx disposal program was initiated [20]. Yet some countries do not have official state guidelines or protocols for the disposal of unwanted and unused medications [21–23]. In Afghanistan, the National Medicine Policy (NMP) emphasizes the disposal of expired medicines by allocating one percent of the cost of all medicines to be provided in Afghanistan, for pharmaceutical product waste management activities. The General Directorate of Pharmaceutical Affairs (GDPA) was held responsible for the systematic monitoring and evaluation of drugs and medical supplies waste management plan implementation throughout the country [24]. Despite these policy recommendations the pharmaceutical waste disposal is facing many shortfalls, and the current system of waste disposal is substantially dysfunctional. To tackle these concerns the Ministry of Public Health (MoPH) formulated Comprehensive Healthcare Waste Management Plan (HCWMP) for the System Enhancement for Health Action in Transition (SEHAT) Project [25] for the handling of pharmaceutical items requiring destruction [26]. These are general policy statements on waste and disposal of pharmaceutical products and lack clear, detailed and transparent procedures [26]. In developing countries like Afghanistan, the inappropriate management of HCW is due to lack of resources, implementation of legislative policies and control [27]. Thus strengthening of policies dealing with HCW disposal, especially in developing economies is needed [5]. In addition, public awareness and different practical approaches are required to dispose unused medications.

No study has so far been conducted regarding disposal practices of unused and expired pharmaceuticals among the general public in Kabul. In Afghanistan, no data is available about the disposal knowledge and practices towards expired medication. This study was therefore planned with the aim to report the current practices and attitudes of general public towards disposal of unused and expired pharmaceuticals.

## Methods

### Study design

This was a descriptive, cross-sectional survey, conducted through face-to-face interviews using pre-validated structured questionnaire. The study was conducted in Kabul between January to March 2016.

### Study population

The study population was of either gender, which included students, public and private sector employees, storekeepers and population from other walks of life, above the age of 18 years, who were local residents of Kabul, regardless of ethnicity or employment status.

### Sampling/sample size

A non-probability sampling technique (convenience method) was employed to reach to the representative population easily in districts four and 10 of Kabul.

### Study instrument

Literature was reviewed to develop the questionnaire [8, 19, 23, 28–32]. The questionnaire consisted of two sections. Section one was about respondent’s personal information including gender, age, marital status, the level of education, ways of procuring medicines, classes of medicine used and checking the expiry date of medicine before procuring. Section two of the questionnaire included respondents’ practices and attitudes concerning unused and expired medication disposal. This included five questions related to the existing unused medicines at their home, what they did with expired and unused medicines, who according to them was responsible to create awareness for proper disposal, and whether improper disposal affected environment and health. The respondents were required to choose from the given descriptions in the questionnaire that best illustrated their usual practices.

The questionnaire was adapted to the local context and translated into the local language and back translated into English to avoid any misinterpretation. For face and content validity, the questionnaire was reviewed by experts and pretested on 15 respondents. Following the pilot testing, minor changes were made based on respondents’ recommendations. For the internal consistency (reliability) assessment, Cronbach’s alpha test was performed.

### Data collection method

The data collectors were trained and prevailed upon to explain the purpose of the study to their potential respondents prior to administering the survey questionnaire. Participation in survey was voluntary. The questionnaire was provided in two languages (Dari and English) to make it more comprehensible. Face-to-face interview method was used by filling up questionnaires.

### Data analysis

All returned questionnaires were double-checked for accuracy and then the collected data were feed into an Excel spreadsheet Dataset. Then the cleaned data was transferred to Statistical Package for Social Science (SPSS) version 23 for analysis. Descriptive statistics (descriptive, crosstab and chi-square) were used.

### Ethical considerations

Written informed consent was obtained from all the respondents before the start of the survey. Participation in this research was voluntary. Participant identity was kept confidential. The Ethical Approval was received from Institutional Review Board of Institute of Public Health under reference number 98092.

## Results

### Demographic data

All the approached 301 individuals agreed to participate in the study and none declined. Thus participant response rate was 100%. Of the 301 respondents, 221 (73.4%) were men and 80 (26.6%) were women. Maximum (104; 34.6%) respondents were aged 32 years and above. One hundred and sixteen (38.6%) respondents had up to secondary education, 163 (54.2%) were university graduates and 22 (7.3%) were illiterate [Table 1]. The Cronbach’s alpha for all items was 0.70.

**Table 1:** Demographics and knowledge about procuring medicines

Variables and categories	Number of responses (%)	
Gender		
Men	221	73.4%
Women	80	26.6%
Age		
18-24	103	34.2%
25-31	94	31.2%
32 – above	104	34.6%
Marital Status		
Single	160	53.2%
Married	141	46.8%
Level of Education		
Illiterate	22	7.3%
Primary	45	15%
Secondary	71	23.6%
University	163	54.2%
Ways of Procuring Medicines		
Purchased on prescription	251	83.4%
Purchased over the counter	44	14.6%
Received from friend/ colleague	3	1%
Purchase based upon the advice of a relative or friend	3	1%
Classes of medicine used		
NSAIDs	61	20.3%
Antibiotic	140	46.5%
Anti-hypertensive	42	14%
Anti-diabetic	23	7.6%
Other	35	11.6%
Do you check expiry date of the medicines before procuring		
Yes	292	97%
No	5	1.7%
Don’t know	4	1.3%

#### Knowledge about procuring medicines

**Fig. 1:**
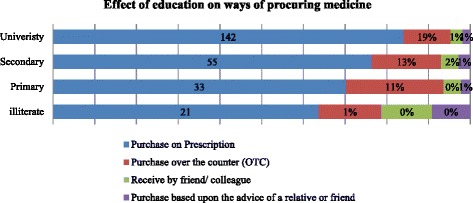
Effect of education on ways of procuring medicine

Regarding knowledge about “ways of procuring medicines”, 251 (83.4%) respondents purchased medicines on prescription and 44 (14.6%) purchased medicine over the counter. Commonly purchased medicines were antibiotics (*n* = 140; 46.5%), NSAIDs (*n* = 61, 20.3%), anti-hypertensive (*n* = 42; 14%), and anti-diabetic (*n* = 23, 7.6%). The majority of respondents (*n* = 292; 97%) checked the expiry date of medicines, prior to purchase [Table 1]. In addition, it was observed that 142 university graduates procured medicines on prescription. Similarly, 159 university graduates checked medicine expiry date prior to purchase [Fig. 1].

Table 2 shows that among all respondent the majority (159/301) of university graduates checked the expiry date of any medicine prior to its procurement.

**Table 2:** Respondents views about checking expiry date of the medicines before procuring

	Do you check expiry date of the medicines before procuring		
	Yes	No	Don’t know
Illiterate	19	1	2
Primary	43	2	0
Secondary	71	0	0
University	159	2	2
Total	292	5	4

In addition, most of the university graduates (*n* = 159) said that improper disposal of unused and expired medicines can affect both the environment and health (Table 3).

**Table 3:** Effect of Education on attitudes towards the effects of improper disposal of unused and expired medicines on environment and health

Level of Education	Yes	Don’t Know	Total
Illiterate	22	0	22
Primary	44	1	45
Secondary	70	1	71
University	159	4	163
Total	295	6	301

#### Practices and attitudes towards unused and expired medication disposal

Table 4 shows responses to the items intended to measure public practices and attitudes towards unused and expired medication disposal and its environmental impact. When asked, did any quantity of purchase medicine remain unused at their home, majority of the respondents (*n* = 287; 95.3%) replied positively. A slim majority (52.2%) of the interviewed respondents kept the unused medicines at home until expired. Most of the respondents (*n* = 234; 77.7%) were throwing the expired medicine in household trash. Six out of 10 (60.8%) of the respondents said that government was responsible to create awareness for proper disposal of unused and expired medicines. A large majority of the sample (*n* = 295; 98%) reported that improper disposal of unused and expired medicines can affect the environment and health. More than 95% of the respondents had unused medicine stored at home, and most of these were antibiotics.

**Table 4:** Respondents’ practices and attitudes concerning unused and expired medication disposal

Questions		N	%
Did any quantity of purchase medicine remain unused at your home?	Yes	287	95.3
	No	14	4.7
What do you do with the unused medicines?	Throw away in household garbage	43	14.3
	Donate to hospital	29	9.6
	Give to friends or relatives	4	1.3
	Return to medical stores	64	21.3
	Keep at home until expired	157	52.2
	Flush unused medications in toilet or sink	4	1.3
What do you do with the expired medicines?	Throw away in household garbage	234	77.7
	Flush expired medications in toilet or sink	36	12
	Give to friends or relatives	4	1.3
	Return to medical store	22	7.3
	Don’t know	5	1.7
Who is responsible to create awareness for proper disposal of unused and expired medicines?	Government	183	60.8
	Pharmaceutical Industries	36	12
	Public	17	5.6
	Pharmacist	65	21.6
Improper disposal of unused and expired medicines can affect the environment and health.	Yes	295	98
	Don’t Know	6	2

## Discussion

Currently medicine waste management and disposal is a hot topic grabbing attention because it has been realized that improper disposal can contaminate the environment and pose the risk to water, air, agricultural products, and food chain and even harm animals/ livestock. Therefore, studies have been conducted throughout the world about this issue to find the policy solutions. However this is the first study from Kabul, Afghanistan.

The Table 1 shows that majority of the respondents purchased medicines on prescription, which shows rational medicine purchase practices. Results from unused medication collection program in California showed that more than 50% of OTC medicines were discarded unused, compared to 45% of prescription medicines [9]. Of total sample, nearly 50% procured antibiotics and the rest procured other classes of medicines such as NSAIDs, anti-hypertensive and anti-diabetic. In procuring medicines the educational level of the respondents played the crucial role, where 142 university graduates purchased medicines on prescription and in contrast only 19 purchased them OTC for self-medication. This indicates that respondent education status correlated with level of awareness of the potential health risks associated with OTC purchase of medicines which has been shown to be taking of excessive drug dosage, continuous medicine use, polypharmacy and drug interactions [33].

Interestingly, in this study nearly all respondents checked the expiry date of medicine, prior to its purchase, while in contrast in the Indian state of Gujarat many were not aware of the expiry date of medicines [34]. It is of paramount significance that prior to purchase or use of any medicine, the expiry date must be checked, otherwise it may lead to serious harmful effects [34].

The current study shows that practices towards disposal of unused and expired pharmaceutical were optimal but more than 95% of the respondents surveyed had left-over medications at home and half of the interviewed population kept the unused medicines at home until expired, which is a source of potential health threat [35]. Borrowing and sharing of medications is known to be associated with several risk factors such as polypharmacy and multiple, chronic comorbidity [36], however, this attitude was rarely observed among the respondents of this study.

The majority of survey respondents asserted, that they throw away the expired medicines in the household trash which is similar to findings of others [23]. Housewives in Busan city of Korea disposed unused medications using the standard garbage bag [37]. Previously it was believed that proper method of unused or expired medications disposal was to flush down the toilet / drain, as opposed to discarding them in the trash, where animals or humans would be more likely to encounter them [38]. More than 10% of the respondents flushed the expired medications down the toilet or sink, which are similar to the practices followed by the people in Kuwait, UK and USA [22, 39], where it is the best practice for liquid medications [23]. Some of the respondents returned unused and expired pharmaceuticals to medical stores, which is similar to community practice in the USA and Malaysia [32, 39]. All these approaches towards safe disposal of leftover pharmaceuticals play significant role in reducing the introduction of pharmaceuticals to the environment since it can cause environmental, human health, and safety hazards [28, 40].

Clear guidance about disposal of unused and expired medicines is lacking and there is a knowledge and practice deficit in appropriate methods for medicines disposal. The Nebraska Medication Education for Disposal Strategies (MEDS) has suggested the “golden standard” for safe, legal, environmentally sound disposal, to put tamper-resistant boxes in pharmacies that will allow consumers to bring medicines back to knowledgeable pharmacists [40]. In Sweden and Korea, more people return unused medicines to a pharmacy for correct disposal, as they have realized the environmental concerns posed by expired medicines [29, 37]. Thus, the Afghan government needs to be proactive to launch feasible expired pharmaceutical collection programs, such as Francisco’s Safe Medicine Disposal program, Dispose a Med program, Chemical Control Program, Sharps Waste Disposal Program Expired medications drop-off operation in California USA [28] Medications Return Program or Take-back programs in Canada [41] and Meds Disposal in Europe [42, 43]. However, if a take-back program is unavailable, household trash is possible as recommended by White House Office of National Drug Control Policy (ONDCP). Prescription medicines along with the patient information should be removed from their original containers and mixed with some undesirable substance, such as kitty litter, coffee grounds, or sawdust inside a sealable plastic bag container and disposed in the trash [44].

The American Pharmacists Association recommends that unwanted medications be crushed or dissolved in water prior to mixing with the undesirable substance [45]. Health care organizations such as Connecticut Department of Environmental Protection suggest four easy steps for medication disposal: First; keep medication in its original container and cross out patient’s name or remove the label. Second; modify the medications to discourage consumption, for example for pills or capsules: add a small amount of water to partially dissolve them; for liquid medications: add salt, flour, charcoal, kitty litter or powdered spice to make a pungent, unsightly mixture that discourages anyone from eating it; for blister packs: wrap pack containing pills in multiple layers of duct tape. Third; seal and conceal and tape medicine container cover closed with packing or plastic tape and then put inside opaque plastic bag or container. Precautionary, the medicines should not be concealed with food items because animals could accidentally eat or drink them. Finally; discard the container in the trash [46].

Despite the suggested alternatives, the proper and best option for the safe disposal of pharmaceutical waste is incineration which requires third party intervention for the collection of unwanted medicines [47]. For example, in Australia, the return of unused medication service runs a medication collection and destruction service through community pharmacies which employ a high-temperature incineration method approved by the US Environmental Protection Agency [23].

Thus, in order to orient the community on proper and standard disposal practices, it is important to increase awareness and undertake training interventions among the public by the government, pharmacists, and pharmaceutical industries. A significant role can be played by community pharmacist being on the forefront in guiding and providing proper education and awareness to the community [34]. Therefore, it is equally essential that their knowledge of proper medication disposal is current, complete, and accurate. Currently, about 20% of pharmacists report learning about medication disposal during pharmacy school [48]. In Taiwan, an educational pharmacist intervention booklet has been designed to teach their customers in how to use and store the medications properly [49]. It is important for the government to focus on the medicines provided free at public hospitals, since it has been shown that free availability of medicine is associated with higher medication wastage. This result is significant in terms of national policy development around medication supplies, and targeting medication wastage [50].

The study results suggest that government, pharmacist, and pharmaceutical industry are responsible to create awareness, which is consistent with the suggestions made by others [32]. Prior to implementing any drug take-back program, stronger campaign and significant involvement of the patient, healthcare professionals and the government officials is required to avoid any possible barriers such as lack of information and techniques of proper disposal of expired medication [51].

It is found that most of the respondents were aware of the hazardous environmental and health impact of improper disposal of unused and expired medicines, which is similar to the concerns of Serbian people [30]. Since the active pharmaceutical ingredients (API) in medicines could be unsafe to the environment, and incorrect disposal of medicines will contaminate the environment [52, 53]. The presence of pharmaceuticals even in small quantities potentially harms aquatic life [54]. A survey from Afghanistan has predicted environmental impact of pharmaceutical waste to be “very low” since, usage of antineoplastic/ cytotoxic preparations, poisons, and hormonal contraceptives in Afghanistan remains low. It further states that a high enough volume of pharmaceutical waste is not currently being generated to create a significant environmental or waste management problem [26].

Bias in medicine disposal practices has been observed due to lack of proper awareness, therefore, there is a pressing need for raising public awareness on proper disposal of unused and expired pharmaceuticals at home and hospitals [29]. In addition, the establishment of a national policy and a legal framework, training of personnel, is essential in successful pharmaceutical waste management [18].

## Conclusion

To further strengthen the ongoing debate on safe disposal practices of unused and expired pharmaceuticals among general public, this survey from Kabul in Afghanistan suggests that, the government is responsible to devise a robust safe and cost effective pharmaceutical waste management program and to make the people aware of hazardous effects of expired and unused medications through comprehensive media campaign. Moreover, like other healthcare practitioners, and community pharmacists are in an excellent position to educate patients on medicine disposal, therefore leveraging their knowledge through training programs and continuous education is of importance.

## Abbreviations

DUMP: Disposal of unwanted medication properly
GDPA: General Directorate of Pharmaceutical Affairs
HCWM: Health Care Waste Management
HCWMP: Comprehensive Healthcare Waste Management Plan
NMP: National Medicine Policy
SEHAT: System Enhancement for Health Action in Transition
WHO: World Health Organization
