# Erratum to: Disposal practices of unused and expired pharmaceuticals among general public in Kabul

**DOI:** 10.1186/s12889-017-4139-5

**Published:** 2017-03-06

**Authors:** Mohammad Bashaar, Vijay Thawani, Mohamed Azmi Hassali, Fahad Saleem

**Affiliations:** 1 Health Policy Analyst, SMART Afghan International Trainings & Consultancy, Kabul, Afghanistan; 2 Professor of Pharmacology, People’s College of Medical Sciences & Research Centre, Bhanpur, Bhopal 462037 India; 3 Professor of Social and Administrative Pharmacy, School of Pharmaceutical Sciences, Universiti Sains Malaysia, 11800 Minden, Penang Malaysia; 4 Faculty of Pharmacy and Health Sciences, University of Baluchistan, Quetta, Pakistan

## Erratum

After publication of the original article [1] it was brought to our attention that author Mohammad Bashaar was incorrectly included as Mohammadk Bashaar. The correct spelling of the name is included in the author list of this erratum and updated in the original article.
